# Developing tiny-sized particles, different modification behaviors of gold atoms, and nucleating distorted particles

**DOI:** 10.1039/d3na00346a

**Published:** 2023-06-30

**Authors:** Mubarak Ali, I.-Nan Lin

**Affiliations:** a Department of Physics, COMSATS University Islamabad Islamabad Campus, Park Road 45550 Pakistan mubarak74@mail.com mubarak60@hotmail.com; b Department of Physics, Tamkang University Tamsui District New Taipei City 25137 Taiwan inanlin@mail.tku.edu.tw

## Abstract

The study of tiny-sized particles is beneficial in many ways. This has been the subject of many studies. The development of a tiny-sized particle depends on the attained dynamics of the atoms. In the development process of a tiny-sized particle, gold atoms must deal with different modification behaviors. Photons traveling along the air–solution interface also alter the characteristics of a developing tiny-sized particle. The electronic structures, modification behaviors, and attained dynamics of the atoms mainly contribute toward the development of tiny-sized particles. Energy under the supplied source and the local resulting forces collectively bind gold atoms. Both internally and externally driven dynamics influence the development process of different tiny-sized particles. Atoms in such developed tiny-sized particles do not experience the collective oscillations upon photons traveling along the air–solution interface. In the study of binding atoms, it is essential to consider the roles of both energy and force. Here, the development of tiny particles having different sizes presents a convincing discussion. Nucleating a distorted particle from the non-uniform amalgamation of tiny-sized particles is also discussed.

## Abstract 2

Magnified HR-TOM image of different tiny-sized particles; distance between the copper capillary and solution surface ∼ 4.0 mm, the concentration of gold precursor ∼ 0.30 mM, and the time of processing solution ∼ 5 min.

## Graphical abstract

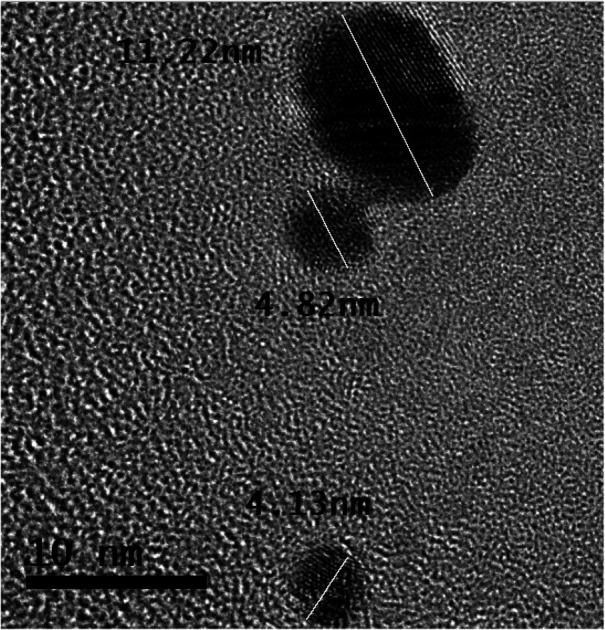

## 1. Introduction

Processing matter at the nanoscale requires new approaches. The study of tiny-sized particles opens applications in various fields.

The study of tiny-sized particles enables us to understand the binding mechanisms in atoms with different properties. In addition to catalytic and photonic applications, the study of tiny metallic colloids has many applications. The beauty of tiny-sized particles is that they can be directly applied in some cases.

The study of tiny-sized particles is a continuous process. Tiny clusters can be used in a wide range of applications.^1^ The stability of nanocrystals suggests that there are methods to fabricate advanced materials with controllable characteristics.^2^ When photons travel along the interface, atoms of tiny-sized particles collectively oscillate.^3^ The development of nanoscale devices is the ultimate long-term goal of nanoparticle technology.^4^ With the successful assembly of a larger particle, the tiny particles can be treated as atoms and molecules for tomorrow's materials.^5^

Understanding dynamics in the development of tiny-sized particles is vital before assembling tiny-metallic colloids for a larger particle.^6^ The assembly of nanoparticles into high-order structures is possible by achieving precise control over the surface properties.^7^

Smaller clusters have molecular-like electronic structures and non-fcc geometric structures.^8^ Several methods to develop tiny-sized particles are available from the literature.^9–12^ The ability to structure matter in the region of sub-optical wavelengths can deliver unusual optical properties.^13,14^ The catalytic activity of metallic nanostructures is significantly enhanced to control the phase transition.^15,16^ Because the charge dynamics are revealed, visualizing and observing an atom in high resolution enables us to understand its functionalities.^17,18^ Under a tuned pulse ON/OFF time, many tiny-sized particles were developed in the shape of an equilateral triangle.^19–22^ The current study discusses the development of different tiny-sized particles in distorted or spherical shapes.

There are quite a large number of methods for processing gold solutions. The literature primarily focuses on plasma sources and green strategies for processing solutions. To synthesize tiny-sized particles, a pulse-based process is a versatile method.

The pulse-based method can synthesize tiny-metallic particles with different features. Some earlier studies have discussed the advantages of the pulse-based process,^19–22^ and it is a cost-effective method that is easy and robust. It can process all sorts of solutions under a variety of parameters, and it is possible to synthesize tiny-sized particles, nanoparticles, and particles having different features.

This pulse-based method of processing the solution is new, where the features of different metallic colloids can be achieved. The development of tiny-sized particles from any method can take advantage of the presented work here, as the underlying science of developing tiny-sized particles in any method remains nearly the same.

This study highlights the fundamental process of developing a variety of tiny-sized particles. This work discusses the electronic configurations, modifications, and dynamics of the atoms. Furthermore, an amalgamation of tiny-sized particles for nucleating a distorted particle is discussed in this study.

## 2. Experimental details

**Fig. 1:**
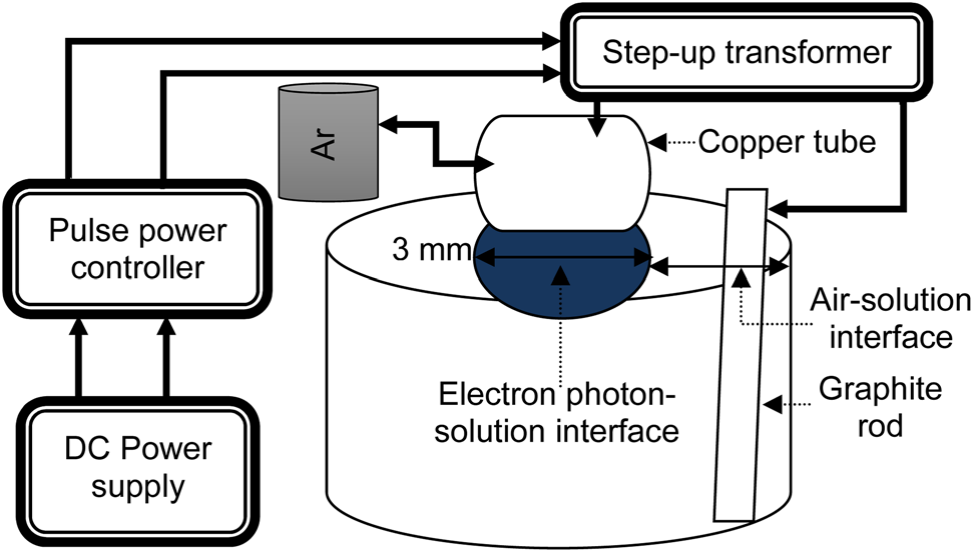
A schematic illustration of the setup.

One method of developing tiny-sized particles is the pulse-based electron–photon–solution interface process. Gold(iii) chloride trihydrate is mixed with DI water to prepare a 100 ml solution in each experiment. A pulsed DC power controller (SPIK2000A-20, MELEC GmbH Germany) was employed to generate and control the bipolar pulses. Fig. 1 shows the layout of the processing method.

A pulse ON/OFF time of 10 μs was set to process the 100 ml solution for 30 s, 1 min, and 5 min. In one experiment, the precursor concentration was 0.2 ml. In the other two experiments, the precursor concentration was 0.3 ml. An argon gas flow rate of 100 sccm was kept constant in each experiment.

The graphite rod is immersed in the solution, and serves as the positive terminal or anode. A copper tube serving as the negative terminal or cathode is adjusted just over the solution surface. At the solution surface, light appeared from the bottom of the copper tube. The splitting of inert gas atoms into electron streams is controlled by the pulse DC power controller. The voltage was ∼31 volts, whereas the current was ∼1.2 amperes. The voltage was enhanced by 40 times *via* the step-up transformer.

Fig. 1 shows the zones of the air–solution and electron–photon–solution interfaces. Further details of the process are given elsewhere.^19^ The diameter of the space of electrons and photons leaving the bottom of the copper capillary becomes slightly larger upon contact with the solution surface. The diameter inside of the hollow space is the internal diameter of the copper tube, which is ∼3 mm. In this method, the energy source is controlled by the tuned pulses.

However, that is not the case in traditional photosynthesis or electrochemical methods. High-resolution transmission optical microscopy (HR-TOM), Model JEOL JEM2100F (also known as HR-TEM) is used to capture the images of the tiny particles.

## 3. Results and discussion

**Fig. 2:**
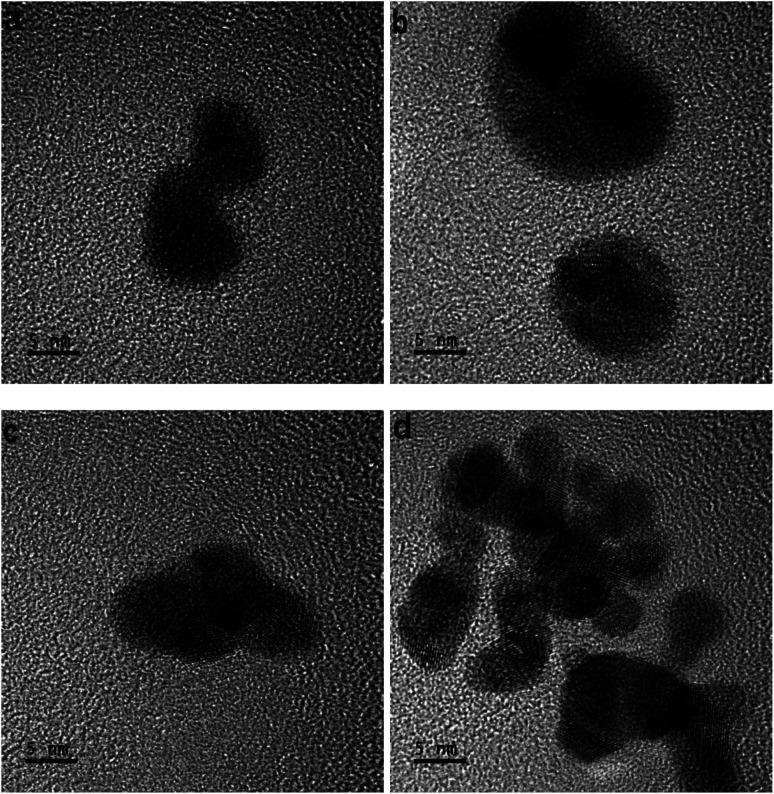
(a–d) HR-TOM images of tiny particles; distance between the point of generation of the light and solution surface ∼ 4 mm, the concentration of the gold precursor ∼ 0.20 mM, and the time of processing solution ∼ 30 s.

In the development of each tiny-sized particle shown in various HR-TOM images of Fig. 2(a)–(d), the atoms do not uniformly amalgamate. The atoms also do not adhere side by side to develop a geometric or anisotropic tiny particle.

The attained dynamics of the atoms are the cause of their amalgamation. A tiny-sized particle developed due to the amalgamation of the gold atoms. In different images of HR-TOM, tiny-sized particles retained different shapes. As a result, they amalgamated non-uniformly and distorted particles nucleated from their non-uniformly attained dynamics. The nucleation of the geometrical or anisotropic-shaped particle follows a different mechanism, which is discussed elsewhere.^19^

In Fig. 2(a)–(d), the atoms develop isotropic or distorted tiny particles. The resulting tiny-sized particles will also develop distorted nanoparticles or particles. Fig. 2(d) shows the amalgamation of many tiny-sized particles nucleating non-uniformly into a distorted particle.

A tiny particle of triangular shape dealing with the localized gravity and levity at the solution surface is discussed elsewhere.^23^ A tiny-sized particle is smaller than a nanoparticle and a particle.^24^ When a tiny-sized particle is evolved instead of developed, the atoms deal with conservative forces at the electron level.^25^

In Fig. 2(a)–(d), different tiny particles developed under the non-uniform amalgamation of atoms. In some parts of the tiny-sized particles, the atoms do not elongate uniformly, so the clamped energy knots to the electrons do not stretch unidirectionally.

A study given elsewhere^26^ discusses the modification behaviors of a gold atom. Atoms of the tiny-sized particles show deformation in different parts. When the atoms do not bind to shape a geometric tiny-sized particle, they also do not elongate. If there is a geometric shape, the arrays of atoms convert into structures of smooth elements.^23^Fig. 2(d) shows the gold atoms amalgamated under significant dynamics. In the amalgamation of gold atoms, suitable forces contribute.

**Fig. 3:**
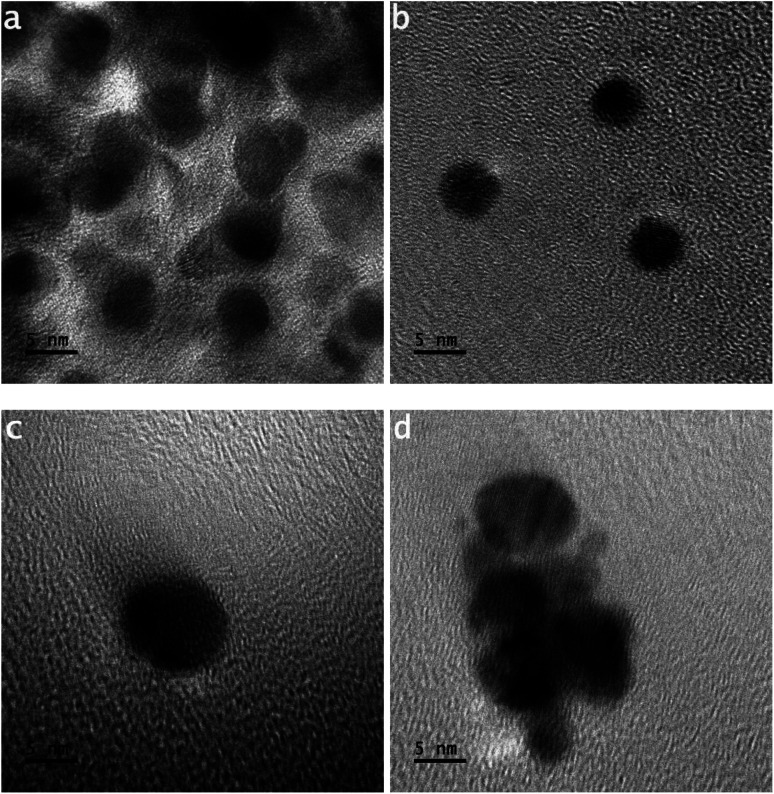
(a–d) HR-TOM images of different tiny-sized particles; distance between the copper capillary and solution surface ∼ 4.0 mm, the concentration of the gold precursor ∼ 0.30 mM, and the time of processing solution ∼ 1 min.

In Fig. 3(a), tiny-sized particles have shapes like an ellipse. Fig. 3(a) shows a large number of tiny-sized particles. The size of each tiny particle is ∼5 nm. In the development of tiny-sized particles, the supplied packets of nano energy do not bind atoms into triangular shapes.

Fig. 3(b) shows three tiny-sized particles of the same shape. The size of each tiny particle is ∼5 nm. The electron streams of the splitting argon atoms impinge on the gold atoms. A tiny-sized particle is smaller than a nanoparticle, whereas a nanoparticle is smaller than a particle.^19,21,22,24^

The influence of the traveling photons on the atoms of a tiny particle having a triangular shape is discussed elsewhere.^23^ In this study, the photons traveling along the air–solution interface also influence the atoms of the tiny-sized particles.

Atoms of tiny-sized particles can elongate or deform. When atoms deform, tiny particles do not form arrays of atoms. Further detail on the different modification behaviors of a gold atom has been discussed elsewhere.^26^

Fig. 3(c) shows a tiny particle having a length of around 8 nm. This loss of resolution in the image is related to the transitional stage image. An HR-TOM image that loses resolution can result when the sample stage vibrates.

At the instant of exposure of the featured photons in the HR-TOM operation, the reading features of the location of the sample provide the information to the output end in shaking or vibrating mode, as shown in the HR-TOM image in Fig. 3(c).

Fig. 3(d) shows the amalgamation of tiny-sized particles under different modes of attained dynamics. In Fig. 3(d), the amalgamating tiny-sized particles are in the phase of nucleating a bigger-sized particle. The amalgamating tiny-sized particles do not keep the same shape and size. As a result, their amalgamation follows the non-uniformly attained dynamics.

**Fig. 4:**
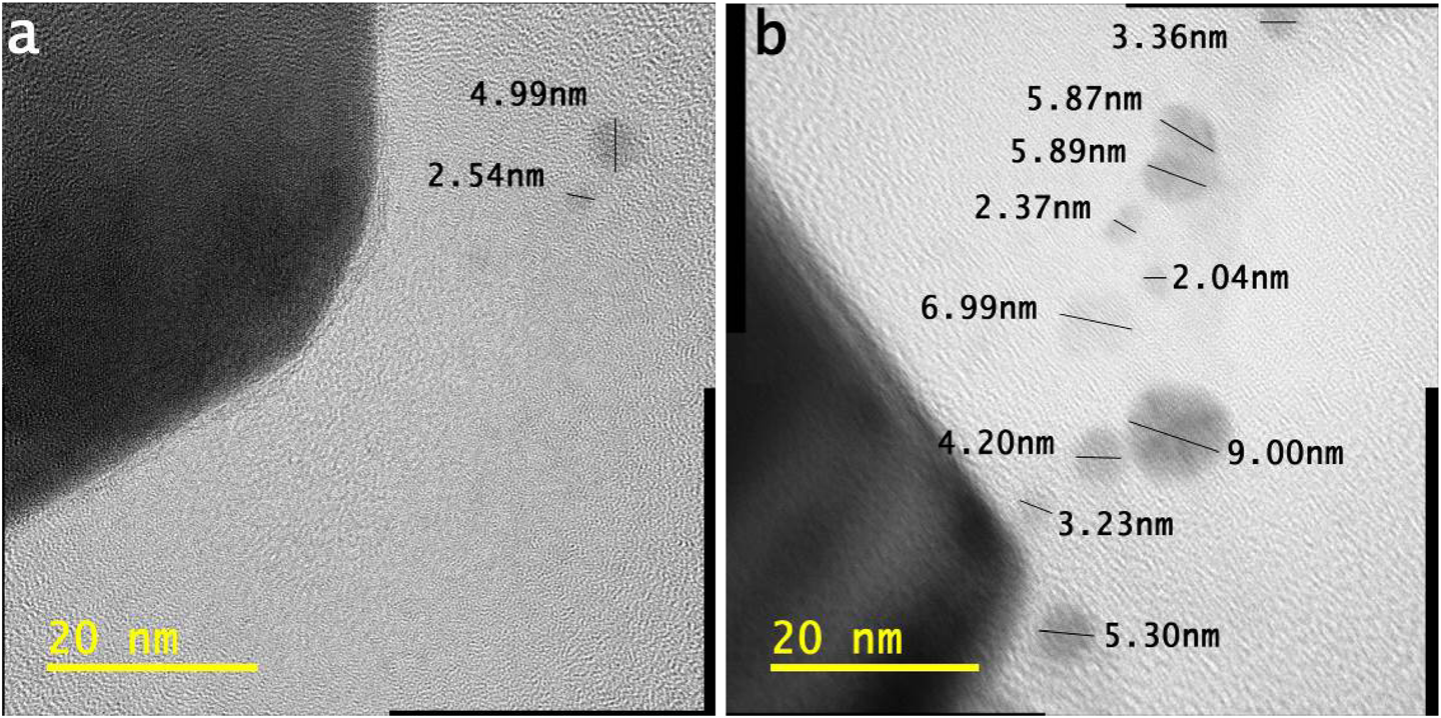
(a and b) HR-TOM images of different tiny-sized particles; distance between the copper capillary and solution surface ∼ 4.0 mm, the concentration of the gold precursor ∼ 0.30 mM, and the time of processing solution ∼ 5 min.

Fig. 4(a) and (b) show tiny particles of different sizes. In both HR-TOM images, the tiny particles retain their size of <10 nm. As per the characteristics of these tiny-sized particles, they can emerge in different applications. In these tiny-sized particles, the atoms modify the structure to develop.

**Fig. 5:**
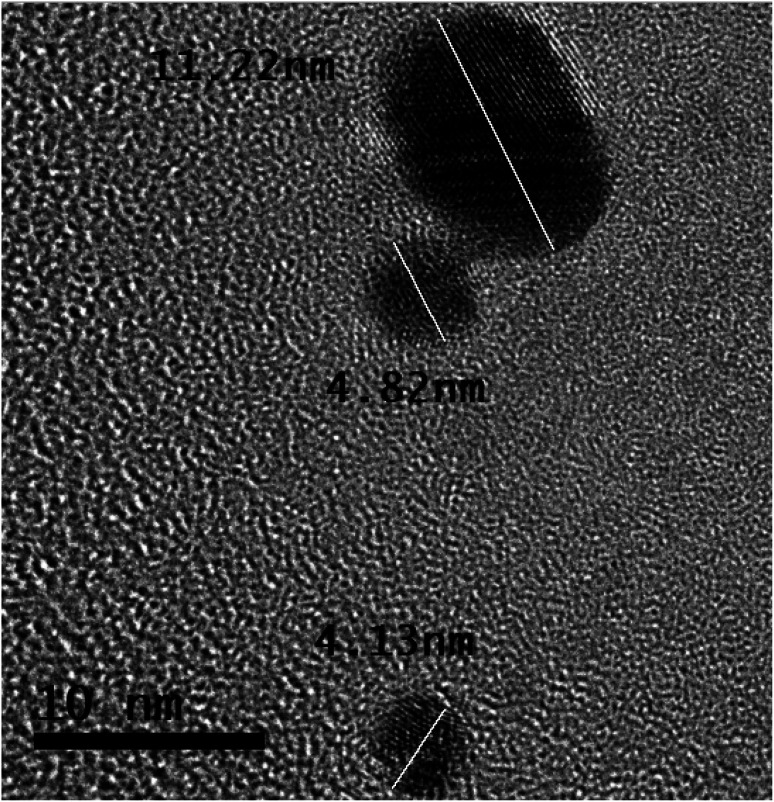
Magnified HR-TOM image of different tiny-sized particles; distance between the copper capillary and solution surface ∼ 4.0 mm, the concentration of the gold precursor ∼ 0.30 mM, and the time of processing solution ∼ 5 min.

In Fig. 5, the half-portion of the tiny-sized particle (length ∼ 11.22 nm) retains the uniform width of each structure of the smooth element, which is ∼0.12 nm.

In Fig. 5, the tiny particle length of ∼11.22 nm shows many distorted atoms. It keeps half of the portion under different stresses. The atoms also validate different configurations of electronic structures.

It is evident from the bigger tiny particle (length of ∼11.22 nm) in Fig. 5 that some structures of smooth elements are fully linear in shape. The photons traveling along the air–solution interface influence the atoms in terms of their order. In Fig. 5, a larger tiny particle clearly shows the different shapes of the atoms.

Some atoms, from the outer sides of the tiny-sized particle, retain blurred shapes. The binding of several layers can be observed in the portion of stress. However, the tiny-sized particle retains the structures of smooth elements in the half-portion. The atoms follow different electronic orientations, indicating their modifications.

Further details on the developing structures of smooth elements and traveling photons along the interface are present elsewhere.^23^ In Fig. 5, the tiny particle with a length of ∼4.82 nm also retains stresses. Most of the atoms indicate a distorted electronic structure.

A tiny-sized particle with a length of ∼4.13 nm in Fig. 5 shows structures of smooth elements in the half-portion. The smaller tiny particles show a different electronic configuration than the bigger tiny particle in Fig. 5.

In the synergy process, the atoms interact with different entities and beaker walls. When the tiny particles develop inside the solution, the atoms do not deal with significant elongation. The surface force exerted on the electrons is less pronounced.

The nucleation time of the discussed tiny-metallic colloids can be less than 1 s or on the order of a few s. Increasing time increases the number of tiny-sized particles, nanoparticles, and particles. However, earlier-developed metallic colloids of any size encounter more interactions with each other and with the walls of the beaker by increasing the processing time. More work is required to study this parameter.

When the tiny-sized particle or molecule deals with the transition state along the reaction path, the atoms also deal with the transition state along the reaction path. Hence, the specific phase transition of an atom is due to the different forces and energy of electrons. Triangular shaped tiny particles developed under the supply of nano energy packets are discussed elsewhere.^23^

**Fig. 6:**
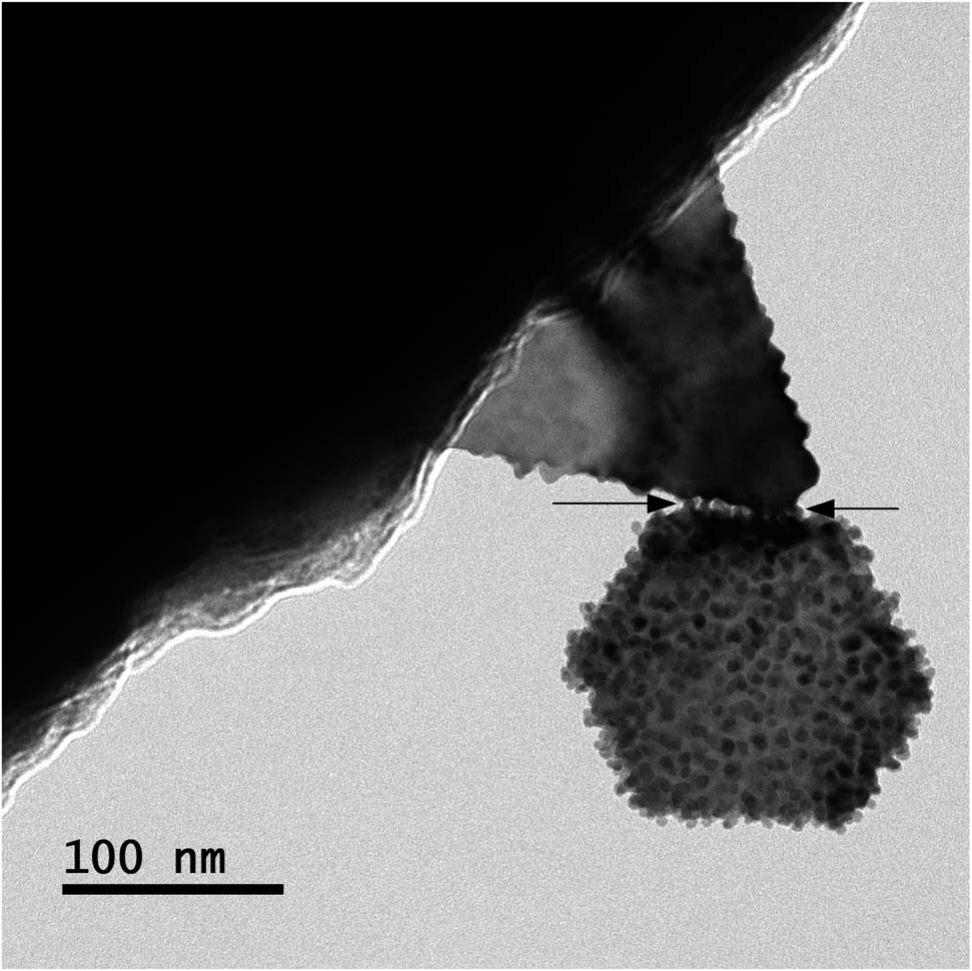
BF-TOM image of two large-sized particles; distance between the copper capillary and solution surface ∼ 4.0 mm, the concentration of the gold precursor ∼ 0.30 mM, and the time of processing solution ∼ 1 min.

Gold atoms bind under suitable force and energy. Energy is contributed at atomic level binding. Electrons of the outer rings or valence rings contribute in atomic binding. From Fig. 6, one can observe that the energy is also contributed at nanoscale level binding, in addition to the micro level and bulk level. In Fig. 6, two large-sized particles cannot bind in such a manner under the influence of only force, so the particles are immature. Before completing the diffusion process of the tiny-sized particles, the process stopped in this case. Thus, those particles could not become faceted and smooth in their shapes. However, further investigations on the coalescence of tiny-sized particles are needed to understand the overall picture. In Fig. 6, the arrows point out the region of binding where the tiny-sized particles bound two large-sized particles.

However, there is a need to investigate the nature and behavior of energy at all scales of binding. There is also a need to investigate the nature and behavior of force at all scales of binding. Gold atoms are in a transition state when binding to develop the tiny-sized particle. Due to the supply of the transition energy, atoms undertake different transition states.^27^ Semisolid atoms execute interstate electron dynamics to generate photon energy.^28^ That photon energy works as photonic current while propagating in the interstate electron gap structure.^26^ That photonic current was the input source for developing many high-aspect ratio nanoparticles and particles.^29^ The pioneering investigations regarding the binding atoms in solution perhaps partially obey the mechanism, as discussed elsewhere.^30,31^

Both force and energy are needed in the binding of atoms. However, carbon atoms obey different mechanisms of binding, as discussed elsewhere.^32^ The Keesom, Debye, and London dispersion forces fall under van der Waals' interactions. These forces are not sufficient to explain the binding of atoms. The role that the van der Waals' forces play in atomic binding does not fully justify the binding mechanism.

It is essential to understand why the role of the van der Waals' forces in atomic binding only partially justifies the binding mechanism, rather than fully.

In developing a tiny-sized particle, both energy and force bind the atoms. Studies like energy modeling are required in this area. Such kinds of investigations certainly extend the area and provide further opportunities.

The nature of force varies depending on the type of atoms. Similarly, the kind of energy changes depending on the atomic nature.

The conserved, partially conserved, or non-conserved behavior of force needs to be addressed depending on the atomic nature and type of binding mechanism.

Our results suggest that the interaction of photons with the atoms of a tiny-sized particle does not produce their collective oscillations. According to this phenomenon, photons travel along the air–solution interface, but the traveling photons neither trap nor couple with the tiny particle. Preliminary details regarding how the van der Waals' interactions and surface plasmons do not support the physical and chemical phenomena are also given elsewhere.^22,23^

In a beaker, gold atoms undertake different modification behaviors. In the development of tiny-sized particles, gold atoms undertake different configurations of electronic structures. A beaker-containing solution has internal parameters, as well as external parameters. A process of synergy depends on both external and internal media. The external medium of the beaker mainly depends on photons and electron streams coming from the light or plasma glow.

In developing a tiny-sized particle, the internal and external media of the beaker both contribute to the process. Both media ultimately contribute to attaining the dynamics of atoms and their modifications. Both external and internal media also influence the tiny-sized particle after development in the solution.

Photons traveling along the interfaces influence the atoms of tiny particles in different ways. A study given elsewhere^23^ discusses the influence of traveling photons along the matter–solution interface. In a beaker, both internal and external media determine the localized dynamics of the process. As a result, the atoms in the solution are influenced by the localized dynamics of the process. There is a need to re-investigate the development mechanisms of different tiny-sized particles.

The study of the interactions of lights or photons with these tiny-sized particles is related to nanophotonics. The traveling photons interact with them to alter their features. The number of photons traveling through the solution that produces this change can be determined by performing further research.

The interactions of photons with developing or developed tiny-sized particles do not validate the collective oscillation of atoms or their lattices. When a tiny-sized particle evolves its structure, it can exhibit this phenomenon. A separate study discusses the surface plasmons phenomenon.^25^

In the presence of different-sized particles, the air–solution interface can affect the number of photons by reflection, refraction, and (or) scattering. More work is required in this area, and the authors believe others will also contribute to this work.

Separate publications are required to report on such topics. A modified atom can be related to the phase transition. However, a phase transition does not infer that the electronic structure of the gold atom is altered. More research is required in this area.

A gold atom mainly deals with the phase transition under the supplied energy. Again, different configurations of electronic structure do not infer that the filled and unfilled states of the gold atom are altered. Atoms retain the perturbed state electrons in the deformation process, so they undertake the non-directional stretching of energy knots in their deformations. Electrons retain no specific orientation.

Keeping the original atomic shape is not possible in developing a tiny-sized particle. To realize a surface plasmon phenomenon, a tiny particle should evolve in structure. Further detail about the surface plasmons is given elsewhere.^25^ Again, protecting the metallic atoms through different capping agents is perhaps not a sustainable way to have the original state.

Changing the distance between the point of generation of the glow and the solution surface changes both the electronic configuration and electronic structure of the gold atoms. As a result, the characteristics of the developing tiny-sized particles and larger-sized particles are altered. A preliminary study given elsewhere discusses the influence of the changing distance between the point of generation of the glow and the solution surface.^19^ However, many studies are required to depict a reliable picture. Such strategies will also help to optimize the experimental parameters to achieve the results for specific applications.

Structural evolutions in atoms of suitable elements are presented elsewhere.^25^ Many studies have discussed tiny particles. Tiny particles other than gold have also been discussed in the literature, and some reports are cited here.^33–40^ The work discussed here presents the science on different topics not previously reported.

The self-organization of tiny-sized particles is due to the balance between the local electrostatic repulsion and dispersion forces.^41^ A gold nanoparticle having a specific number of atoms can be utilized effectively as a catalyst.^42^ A composite structure involving gold nanoparticles was investigated for sensor application.^43^ An energy shift of the gold nanoparticles was observed at different wavelength excitations.^44^ A review study has reported on ligands-protected noble metal nanoclusters for various applications.^45^ A study elsewhere^46^ discussed the scattering properties of the metal-based tiny particles. A gold nanoparticle-based composite shows more effectiveness at nanobubble generation than the current methods of plasmonic nanoparticle cavitation.^47^

A mechanism of the antibacterial activity of Co_3_O_4_ and polypyrrole nanocomposites was proposed.^48^ The development of metal nanoparticles encapsulated with polypyrrole plastic nanocomposites was reviewed.^49^ To examine the antibacterial activities, a variety of complexes was developed in a template reaction.^50^ The mixed metal nanocomposites showed good photocatalytic activity against methyl red dye.^51–56^ Different synthetic approaches and advancements in synthesizing nanomaterials were reviewed.^57^ Those reported nanocomposites, nanomaterials, or quantum dots for various applications are also related to tiny-sized particles.

However, before communicating authentic and sustainable applications, it is essential to understand the fundamental science of developing tiny-sized particles, atomic modification, and nucleating distorted particles. Such proactive measures help to secure a sustainable application of different metallic clusters.

## 4. Conclusions

Depending on the attained dynamics of the atoms, different tiny-sized particles can be developed. In the development process of a tiny-sized particle, the localized force and energy bind the atoms. In addition to the input parameters, multiple factors contribute to the development of different-sized tiny particles. Tiny particles are developed in different shapes depending on the local conditions of the process.

Configuring the electronic structures of the gold atoms differently in the tiny-sized particle validates the role of localized dynamics. A process of synergy determines the modification behavior of the atoms, and also determines the features.

A tiny particle shows different modification behaviors of the atoms. The structures of smooth elements can be distorted due to the process of synergy. Due to the traveling photons along the air–solution interface, a tiny particle can also modify the electronic structures of the gold atoms.

The present work validates that tiny particles do not develop only by force (such as in van der Waals forces), and they also disregard the phenomenon of surface plasmons. A surface plasmon phenomenon is viable in evolving tiny particles instead of developing them.^25^ The developmental process of the tiny-sized particle partially obeys the van der Waals forces to bind atoms since the element of energy is also there. Due to different interactions, an early-developed tiny particle can be distorted.

When tiny-sized particles do not retain a specific shape and size, their amalgamation nucleates into a bigger-sized distorted particle. The study introduces new concepts in both physical and chemical sciences. As a result, the study of tiny-sized particles opens new horizons to science.

## Data availability

Data sharing does not apply to this article, as no new data were created or analyzed.

## Author contributions

Mubarak Ali: conceptualization, investigation, validation, formal analysis, writing – original draft, writing – review & editing, visualization. I.-Nan Lin: supervision, resources.

## Conflicts of interest

The author declares no conflicts of interest.

## Supplementary Material
